# Mental health services for children exposed to armed conflict: Médecins Sans Frontières’ experience in the Democratic Republic of Congo, Iraq and the occupied Palestinian territory

**DOI:** 10.1179/2046905513Y.0000000098

**Published:** 2013-11

**Authors:** K Lokuge, T Shah, G Pintaldi, K Thurber, C Martínez-Viciana, M Cristobal, L Palacios, K Dear, E Banks

**Affiliations:** 1 The Australian National University, Canberra, ACT, 0200, Australia; 2 MSF-UK, London; 3 MSF-Operational Centre, Amsterdam, The Netherlands; 4 MSF-Operational Centre, Barcelona, Spain

**Keywords:** Children, Mental health, Armed conflict, Mental health services, MSF, Adolescents

## Abstract

**Background:** Armed conflict has broad-ranging impacts on the mental health and wellbeing of children and adolescents. Mental health needs greatly exceed service provision in conflict settings, particularly for these age groups. The provision and targeting of appropriate services requires better understanding of the characteristics and requirements of children and adolescents exposed to armed conflict.

**Methods:** Routine patient and programme monitoring data were analysed for patients <20 years of age attending mental health services provided by Médecins Sans Frontières (MSF) in three countries affected by armed conflict: the Democratic Republic of Congo (DRC), Iraq and the occupied Palestinian territory (oPt). The demographic characteristics, presenting mental health complaint, attributed precipitating event, services provided and short-term outcomes for mental health services users in each country are described.

**Results:** Between 2009 and 2012, 3025 individuals <20 years of age presented for care in DRC and Iraq, and in 2012 in oPt, constituting 14%, 17·5% and 51%, respectively, of all presentations to MSF mental health services in those three countries. The most common precipitating event was sexual violence in DRC (36·5%), domestic violence in Iraq (17·8%) and incarceration or detention in oPt (33%). Armed conflict-related precipitants were reported by 25·9%, 55·0% and 76·4% of youths in DRC, Iraq and oPt, respectively. The most common presenting complaints in children and adolescents were anxiety-related, followed by mood-related, behaviour-related and somatisation problems; these varied according to country and precipitating event. Although a high proportion (45·7%) left programmes early, 97% of those who completed care self-reported improvement in their presenting complaint.

**Conclusions:** Brief trauma-focused therapy, the current MSF mental health therapeutic intervention, appears to be effective in reducing symptoms arising from the experience of trauma. Although inferences on outcomes are limited by high default rates, this provides a feasible tool for addressing the mental health needs of children exposed to armed conflict. Priorities for future research include understanding why children and adolescents constitute a small proportion of patients in some programmes, why many leave care early and how to address these issues, but this research must occur within the context of efforts to provide access to mental health services for children.

## Introduction

In the past century, more than 100 million lives have been claimed directly by armed conflict; it is estimated that more than half of these have been children under 5 years of age.^1^ In the past decade alone, 2 million children have died, 4 million children have been injured, and one million children have been orphaned or separated from their families.^2^ Adolescents are vulnerable not only through civilian exposure to the impacts of armed conflict, but often also through active participation as armed combatants.^3^

It is recognised increasingly that, apart from direct physical impact, armed conflict has broad-ranging impacts on the mental health and wellbeing of children and adolescents.^4^ There is an urgent need to provide appropriate mental health services to address this, but requirements in resource-limited settings greatly exceed service provision; it is estimated that, in low- and middle-income countries, between 76% and 85% of people with severe mental disorders receive no treatment.^5^

The situation is even starker in conflict settings.1,^6^^–^9 It has been recognised that the mental health needs of children and adolescents must become an explicit part of any response in conflicts,^8^ but this recognition has yet to translate into effective and accessible mental health services.^10^ Applicable research on mental health interventions for conflict-affected children and adolescents has been very limited^11^^–^13 and, consequently, few organisations have adequate guidelines for their care, including the diagnosis and management of mental health problems.^7^ Developing an appropriate evidence base is a vital pre-requisite to developing such guidelines; an important component of such evidence is to understand the characteristics and mental health care requirements of children and adolescents affected by armed conflict.

## The Médecins Sans Frontières Experience

Médecins Sans Frontières (MSF), founded in 1971, is an international, independent, medical humanitarian organisation that delivers emergency aid to people affected by armed conflict, epidemics, natural disasters and excluded from health care. It is a non-profit, self-governed organisation working in some 70 countries worldwide. As part of its work, MSF frequently provides mental health and psychosocial support for communities affected by conflict.^14^

This paper presents an analysis of routine patient and programme monitoring data on children and adolescents attending mental health services provided by MSF in three countries affected by armed conflict: the Democratic Republic of Congo (DRC), Iraq and the occupied Palestinian territory (oPt).

The aims of this analysis were to: (i) describe the demographic characteristics of children and adolescents presenting to these programmes; (ii) describe the presenting mental health complaints and precipitating or underlying events associated with these complaints; (iii) compare the above factors across the three countries included in this analysis; and (iv) describe the mental health services provided and their short-term outcomes in each country.

## Methods

DRC, Iraq and oPt were chosen for this analysis because they have long-standing MSF-supported mental health programmes which target populations affected by armed conflict. Data were available from each programme for the following periods on all patients presenting for care: DRC and Iraq during 2009–2012 and oPt during 2012. Programmes in each country include projects in the following locations: DRC (Mweso and Kitchanga), Iraq (Fallujah, Imam Ali and Yarmouk) and oPt (Hebron and East Jerusalem).

## Context

### Democratic Republic of Congo

The protracted conflicts in DRC have created an on-going and complex humanitarian crisis, with an estimated 2·3 million displaced people.^15^ The mental health impact of this violence and displacement is considerable,^16^ but the health system in most areas has little or no capacity to respond to these needs.^17^ The Mweso health zone, in North Kivu province, consists of a large and dispersed population with very limited access to healthcare and formal services for mental health, even in times of stability. During unstable and violent periods, displaced populations stay in overcrowded camps with host families or sleep in the fields to avoid overnight attacks. People often walk long distances to obtain provisions and health care, putting them at risk of extortion and further violence. Attacks on civilians are often brutal and include the destruction of homes and villages.

### Iraq

Since 2003, Iraq has experienced significant waves of conflict, terror and violence with a devastating impact on the country’s economy and the health of its population.^18^^–^21 The security and wellbeing of Iraq’s children has been threatened by kidnapping, loss of parents, displacement, malnutrition, deterioration of the education system, religious persecution, child labour, child trafficking and active participation as combatants.^21^ Iraq’s health system relies on a very small health workforce: in 2005, there were only 6·1 physicians per 10,000 population compared with the regional average of 10·8.^22^ The mental health services are even more limited, with around 145 psychiatric nurses, 100 psychiatrists, 25 social workers and 16 psychologists across the entire country.^21^ These resources are incapable of meeting the country’s needs. An estimated 36% of adults suffer significant psychological trauma, predominantly resulting from direct and indirect experiences of violence.^23^ The burden on children and adolescents, who constitute nearly 50% of the country’s population,^24^ is estimated to be even higher.^20^

### Occupied Palestinian territory

The Israel–Palestine conflict has affected Palestinian and Israeli societies for decades, and has reverberated throughout the Middle East. The conflict continues to lead to civilian casualties, displacement and humanitarian needs. The region’s population is around 4·2 million, with only 1·8 primary health care centres per 10,000 population.^25^ The health system is fragmented, and the quality of care has decreased owing to severe shortages of drugs and supplies, unstable power supply and inadequate maintenance capacity,^26^ and referral for appropriate health care is difficult owing to movement restrictions.^27^ Services to address the psychological needs arising from conflict-related violence include a psychiatric hospital in Gaza with a capacity for only 24 patients, another for general psychiatric conditions in Bethlehem, and some community mental health centres, inadequate to treat the estimated 25,000–50,000 people requiring psychological intervention owing to the crisis.^26^

### Programme descriptions

Although all programmes provided mental health services, the model of mental health care delivery differs between countries in terms of human resources, outreach activities and its relationship to other health services. The mental health service in DRC is integrated with several other health activities, including primary healthcare. In Iraq and oPt, the mental health programmes are not integrated with other health services, and the Iraqi programmes are all hospital-based. The DRC programme provides training for community health workers on recognition and referral of patients with mental health problems, and also conducts community outreach activities carried out by MSF, but the focus is awareness-raising around specific issues such as sexual violence. Only oPt has community-based mental health outreach activities specifically targeting children. In DRC and Iraq, programmes are run by staff with basic counselling training who are supported by specialist advisers, whereas in oPt mental healthcare is provided by national and international psychologists. Management (including operational management and technical oversight) of the programmes also differs: the DRC and Iraqi programmes are managed by the Amsterdam Operational Centre of MSF (MSF-OCA), and the oPt programme is managed by the Barcelona Operational Centre (MSF-OCBA).

### Counselling interventions

The counselling approach in all the programmes is based on principles derived from brief trauma-focused therapy as outlined in the MSF mental health guidelines.^28^ In DRC and Iraq, the mainstay of the programme is individual counselling by lay counsellors from the community, whereas in oPt therapy is delivered by psychologists. However, programmes in all three countries have a defined process and goal-orientated outcomes, with the emphasis on observable behaviour change and symptom reduction leading to improved functioning rather than treatment of specific psychiatric disorders.

### Data sources

MSF routinely collects individual-level data on the medical and psychosocial care provided through its programmes. These include basic demographic data such as age, gender and place of residence of patients, information on the self-reported precipitating event(s) associated with presentation, the presenting symptom(s)/complaint(s), the severity of symptoms and functional impairment (rated on a scale from one to 10), data on the patient’s ongoing self-assessment of change in symptoms and functioning until discharge from the programme, and on care provided and self-reported outcomes. On discharge from care, the mental healthcare provider also records their assessment of the status of the presenting complaint.

All data used in this analysis were collected for the purposes of patient care and routine programme monitoring. Data were entered at each programme location into a secure, password-protected electronic database, with patient identification number as the only personal identifier. DRC and Iraq use a common database and coding system for variables; however, data from the oPt programmes had greater breakdown of presenting complaints and symptoms. For these variables, specific events or symptoms described in oPt were assigned by oPt mental health advisers to one of the broader categories used in DRC and Iraq to facilitate comparison between the countries included in this analysis.

### Main precipitating event associated with presentation

The first recorded precipitating event was used in this analysis, although several events could be recorded. In DRC and Iraq, the first recorded event represents the main precipitating event as reported by the patient. In oPt, although the events were not necessarily ranked in order of importance, only the first-recorded was included in the analysis. Specific events were assigned to the following categories:

- Armed conflict: all events associated with intentional incidents of violence perpetrated as part of the conflict, excluding sexual violence (hereafter ‘armed conflict’).
- Sexual violence: includes sexual assault and rape; data in this category were not disaggregated by location or perpetrator, and therefore include sexual violence perpetrated by family members.
- Domestic violence or neglect: physical violence (apart from sexual violence) or neglect between family members.
- Non-violence-related: precipitating events not associated with intentional violence including events such as accidents and schooling difficulties.

### Main presenting complaint

Although several presenting complaints could be recorded, only the main presenting complaint reported by patients in DRC and Iraq, and the first recorded (but not necessarily main) complaint in oPt reported by the patient were used for this analysis.

DRC and Iraq use a common, pre-defined system for categorising symptoms, described in detail in the MSF mental health guidelines.^28^ These categories are based on groups of symptoms related to specific mental health conditions. Symptoms associated with post-traumatic stress disorder (PTSD) are grouped under anxiety-related complaints, and depression-associated symptoms are grouped under mood-related complaints. The remaining categories included behaviour-related complaints and physical complaints (somatisation disorders). Symptoms that did not fit any of these categories were included as ‘other’. The specific symptoms and complaints reported in oPt were assigned to the appropriate category by mental health specialist staff overseeing the oPt programme.

### Complaint rating

The severity of the presenting complaint is rated by the patient on a scale of one to 10 in all three countries. However, in DRC and Iraq, the scale indicates increasing severity from left to right (1 = most severe), whereas in oPt it is the opposite (1 = least severe). Therefore, when calculating change in complaint severity from presentation to discharge, to allow ratings to be compared, the direction of change was transformed from negative to positive in the oPt data. The degree of change remained the same. This rating scale has been used for several years by MSF in a range of settings, and, before use, counsellors verify client understanding of the scale. Previous assessment of this scale has found a strong correlation between patient self-rating using it and counsellor rating of complaint severity across all countries and settings in which it has been used by MSF.^29^

### Statistical analysis

Data were aggregated across sites within each country, and analysed separately for each country. Age distributions were examined initially using data across all patients presenting to the selected programmes, including adults. Following this, analysis of patient characteristics, care provided and outcomes was restricted to those in the age groups <15 years old (hereafter referred to as ‘children’) or 15–19 years old (hereafter referred to as ‘adolescents’). Univariate analyses using *χ*^2^ tests were explored variations in patient and care characteristics by country, age group and gender. No statistical imputation was carried out for missing values; therefore, proportions are reported with a denominator representing the number of individuals with a non-missing value for that factor. The only variable for which a value was assigned to missing data was discharge from care. A patient was considered discharged from care if they were documented as discharged from the programme for that episode by a healthcare provider. If a patient was recorded as leaving before discharge for any other reason, or if a missing value was assigned to their exit category, it was assumed for the purposes of this analysis that the patient left care before discharge.

This review complied with MSF Ethical Review Board requirements for analysis of routinely collected programme data. Ethical approval was also obtained from the Australian National University Human Research Ethics Committee. Patient identification numbers, the only personal identifier recorded in the database, were removed from the dataset before analysis.

## Results

### Age and sex distribution by country

A total of 17,655 individuals of all ages including 1258 children (7·1%) and 1767 adolescents (10·0%) presented for care. The age distribution by country of the patient population including adults is given in Table 1. The proportion of all patients presenting for care who were children varied significantly by country (3·7%, 8·0% and 36·5% in DRC, Iraq and oPt, respectively, *P*-<0·001), as did the proportion of adolescents (10·4%, 9·5% and 14·6% in DRC, Iraq and oPt, respectively, *P*<0·001).

**Table 1:** Age distribution by country of adults and children

	DRC	Iraq	OPT	Total	*P*-value*
0–4 y, *n* (%)	10 (0·1)	48 (0·5)	22 (3·8)	80 (0·5)	*P*<0·001
5–9 y, *n* (%)	45 (0·6)	310 (3·2)	75 (12·8)	430 (2·4)	*P*<0·001
10–14 y, *n* (%)	221 (3·0)	411 (4·3)	116 (19·9)	748 (4·2)	*P*<0·001
15–19 y, *n* (%)	775 (10·4)	907 (9·5)	85 (14·6)	1767 (10·0)	*P*<0·001
≧20 yrs, *n* (%)	6433 (86·0)	7911 (82·5)	286 (49·0)	14,630 (82·9)	*P*<0·001
Total all ages, *n*	7484	9587	584	17,655	
Total <15 y, *n* (%)	276 (3·7)	769 (8·0)	213 (36·5)	1,258 (7·1)	*P*<0·001
Total <20 y, *n* (%)	1051 (14·1)	1676 (17·5)	298 (51·0)	3025 (17·1)	*P*<0·001

* Fischer’s Exact test; DRC, the Democratic Republic of Congo; oPt, the occupied Palestinian territory.

The male-to-female ratio of those presenting for care varied between countries (Table 2) (*P*-<0·001 for variation between countries in proportion of female children and adolescents). The proportion of females presenting in DRC (69% of children, 77% of adolescents) was much higher than in either Iraq or OPT, and OPT had the highest proportion of males presenting (66% of children and 74% of adolescents were male).

**Table 2:** Gender distribution by age group and country for children and adolescents

	Male	Female	Total
Children <15 y*			
DRC, *n* (%)	86 (31·2)	190 (68·8)	276 (100)
Iraq, *n* (%)	438 (57·0)	330 (43·0)	768 (100)
OPT, *n* (%)	141 (66·2)	72 (33·8)	213 (100)
Total, *n*	665	592	1257
Adolescents 15–19 y*			
DRC, *n* (%)	181 (23·4)	594 (76·7)	775 (100)
Iraq, *n* (%)	313 (34·5)	594 (65·5)	907 (100)
OPT, *n* (%)	63 (74·1)	22 (25·9)	85 (100)
Total, *n*	557	1210	1767

* Fischer’s Exact test, *P*<0·001.

### Referral

Those presenting were referred for care from a range of sources (Tables 3A and B). The largest proportion in DRC (42·6%) was referred from primary healthcare providers. Other important sources in DRC were MSF-run community outreach activities (28%), and referrals by secondary and tertiary health services (18%). These were all important sources in Iraq also, although secondary and tertiary health services were the single most common source reported (33%). In Iraq, family and friends referred 18% of patients, but in DRC very few patients presented through this route. In oPt, the most important sources of referral were MSF counsellors themselves (27%), international and local NGOs (25%) and family members (23%). Mass media-related sources such as radio and pamphlets were associated with very few presentations in all programmes.

**Table 3A:** Referral source for children and adolescents in DRC and Iraq

	DRC *n* (%)	Iraq *n* (%)
Primary healthcare: includes referrals from MSF and other providing primary healthcare services (other NGO, MoH)	448 (42·6)	429 (25·6)
Community activities: includes mass education, discussion groups, etc. executed by MSF	293 (27·9)	281 (16·8)
Secondary/tertiary care: referrals by specialised health centre, hospital, psychiatric institution	189 (18·0)	547 (32·7)
Hearsay: friends, neighbours, family recommendation	90 (8·6)	300 (17·9)
Other	24 (2·3)	36 (2·2)
Non-medical organisations: includes referrals by NGOs, local authorities (e.g. social affairs), religious workers, traditional healers	7 (0·7)	22 (1·3)
Mass media: leaflets, radio, posters, etc.	0	60 (3·6)
Total	1051 (100·0)	1675 (100·0)

**Table 3B:** Source of referral of children and adolescents in oPt

Palestine	*n* (%)
MSF counsellor	80 (26·9)
International/local NGOs	74 (24·8)
Family member	69 (23·2)
Self-referral	34 (11·4)
Comments by others	13 (4·4)
Others	10 (3·4)
MSF psychologist/psychiatrist	8 (2·7)
MoH or other health staff (non-MSF)	5 (1·7)
Other MSF staff	2 (0·7)
Community health worker	1 (0·3)
Institutions/church, etc.	1 (0·3)
Knowledge of intake: leaflets/posters	1 (0·3)
Total	298 (100)

### Precipitating events

**Figure 1:**
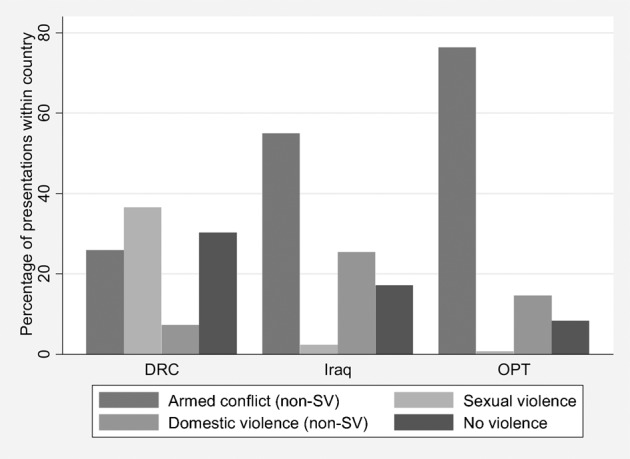
Proportion of children and adolescents <20 y presenting with each category of precipitating event, by country. DRC, the Democratic Republic of Congo; oPt, the occupied Palestinian territory

The proportion of children with a mental health problem associated with armed conflict (other than sexual violence) varied significantly between the countries (27·9%, 58·6% and 75% in DRC, Iraq and oPt, respectively, *P*<0·001) (Table 4 and Figure 1). In Iraq and oPt, the most common reason for presentation was armed conflict, and in DRC it was sexual violence (42% of children and 35% of adolescents, *P*-value<0·001 for variation between countries for both age groups). In Iraq and oPt, very few children or adolescents presented because of sexual violence.

Domestic violence or neglect was also commonly reported by children, but varied by country (4%, 21% and 16% in DRC, Iraq and oPt, respectively, *P*<0·001 for variation between countries) (Table 4). Non-violence-related events precipitating presentation were reported by 18·1% of children presenting overall, and, again, this varied significantly between country (26·8%, 17·7% and 8·2% in DRC, Iraq and oPt, respectively, *P*<0·001). The pattern of these precipitating events was similar for children and adolescents within each country, but there was significant variation in children between countries.

**Table 4:** Precipitating event by age group and country

	DRC	Iraq	OPT	Total	Fischer’s Exact test
*Children (<15 years)*					
Armed conflict (excluding sexual violence)					
*n* (%)	77 (27·9)	450 (58·6)	156 (75·0)	683 (54·6)	*P*<0·001
95% CI	22·7–33·6	55·0–62·1	68·5–80·7	51·7–57·3	
Sexual violence					
*n* (%)	115 (41·7)	18 (2·3)	1 (0·5)	134 (10·7)	*P*<0·001
95% CI	35·8–47·7	1·4–3·7	0·0–2·6	9·0–12·5	
Domestic violence (excluding sexual violence) and neglect					
*n* (%)	10 (3·6)	164 (21·4)	34 (16·4)	208 (16·6)	*P*<0·001
95% CI	1·8–6·6	18·5–24·4	11·6–22·1	14·6–18·8	
Not related to violence					
*n* (%)	74 (26·8)	136 (17·7)	17 (8·2)	227 (18·1)	*P*<0·001
95% CI	21·7–32·5	15·1–20·6	4·8–12·8	16·0–20·4	
Total, *n* (%)	276 (100)	768 (100)	208 (100)	1252 (100)	
					
*Adolescents (15–19 years)*					
Armed conflict (excluding sexual violence)					
*n* (%)	195 (25·2)	472 (52·0)	64 (80·0)	731 (41·5)	*P*<0·001
95% CI	22·1–28·4	48·7–55·3	69·6–88·1		
Sexual violence					
*n* (%)	269 (34·7)	21 (2·3)	1 (1·3)	291 (16·5)	*P*<0·001
95% CI	31·4–38·2	1·4–3·5	0·0–6·8		
Domestic violence (excluding sexual violence) and neglect					
*n* (%)	67 (8·7)	263 (29·0)	8 (10·0)	338 (19·2)	*P*<0·001
95% CI	6·8–10·8	26·1–32·1	4·4–18·8		
Not related to violence					
*n* (%)	244 (31·5)	151 (16·7)	7 (8·8)	402 (22·8)	*P*<0·001
95% CI	28·2–34·9	14·3–19·2	3·6–17·2		
Total, *n* (%)	775 (100)	907 (100)	80 (100)	1762 (100)	

95% CI, 95% confidence intervals.

Within countries, there were significant differences in the male-to-female ratio for different precipitating events (data not shown). In DRC, significantly more female children (98%) and adolescents (91%) presented following sexual violence. In Iraq, significantly more female adolescents presented in all categories of precipitating event. In the armed conflict category in oPt, around two-thirds of all who presented were male children and adolescents.

### Details of presentation for armed conflict (non-sexual-related violence)

Table 5 gives a detailed breakdown of all precipitating events for children and adolescents in the armed conflict category within countries. The commonly reported specific events in DRC in the armed conflict category were psychological violence and physical violence. In Iraq, the common events were psychological violence and witnessing abuse, injury or death (17·8% and 14·4% of all presentations, respectively). The most common precipitating event in oPt was incarceration or detention (27·4% of children and 47·5% of adolescents reported this specific event).

**Table 5:** Detailed description of presentations in the armed conflict category (excluding sexual violence) by country and age group

	*n* (%)	95% confidence interval
**Children <15 y**		
DRC	77 (27·9)	22·7–33·6
Physical violence (intentional)	21 (7·6)	4·8–11·4
Psychological violence (intentional)	17 (6·2)	3·6–9·7
Other armed conflict event	39 (14·1)	10·2–18·8
Total presentations for which event recorded	276 (100·0)	–
Iraq	450 (58·6)	55·0–62·1
Witnessing abuse, injury or death	143 (18·6)	15·9–21·6
Psychological violence (intentional)	116 (15·1)	12·6–17·8
Intentional abuse while in detention	41 (5·3)	3·9–7·2
Other armed conflict event	150 (19·5)	16·8–22·5
Total presentations for which event recorded	768 (100·0)	–
oPt	156 (75·0)	68·5–80·7
Incarceration/detention	57 (27·4)	21·5–34·0
Witnessed violence (physical, killing, threats)	39 (18·8)	13·7–24·7
Received threats	28 (13·5)	9·1–18·9
Other armed conflict event	32 (15·4)	10·8–21·0
Total presentations for which event recorded	208 (100·0)	–
**Adolescents 15–19 y**		
DRC	195 (25·2)	22·1–28·4
Psychological violence (intentional)	48 (6·2)	4·6–8·1
Physical violence (intentional)	34 (4·4)	3·1–6·1
Other armed conflict event	113 (14·6)	12·2–17·3
Total presentations for which event recorded	775 (100·0)	–
Iraq	472 (52·0)	48·7–55·3
Psychological violence (intentional)	182 (20·1)	17·5–22·8
Witnessing abuse, injury or death	99 (10·9)	9·0–13·1
Deprivation and discrimination	44 (4·9)	3·5–6·5
Other armed conflict event	147 (16·2)	13·9–18·8
Total presentations for which event recorded	907 (100·0)	–
oPt	64 (80·0)	69·6–88·1
Incarceration/detention	38 (47·5)	36·2–59·0
Witnessed violence (physical, killing, threats	10 (12·5)	6·2–21·8
Other physical violence (wounded, beaten, tortured)	16 (20·0)	11·9–30·4
Total presentations for which event recorded	80 (100·0)	–

### Presenting complaints

Anxiety-related complaints, including symptoms such as stress, worry and fear, were the most common presenting symptoms in children and adolescents in all countries (33·1%, 38·2% and 32·9% for DRC, Iraq and oPt, respectively, *P* = 0·015) (Table 6). The proportion of children and adolescents presenting within each category (anxiety, mood, behaviour, physical complaints attributable to underlying psychological problems and other) varied significantly between countries (all *P*<0·001). The proportion with mood-related problems was much higher in DRC than in the other countries (32·5% in DRC compared with 10·2% and 11·3% in Iraq and OPT, respectively), and the proportion with physical complaints attributable to underlying psychological problems was much higher in oPt than in the other two countries (39·7% in oPt compared with 14·9% and 17·8% in DRC and Iraq, respectively).

### Commonly reported complaints associated with common precipitating events

Table 7 gives the commonly reported specific precipitating events and common main presenting complaints with which they were associated in each country. In DRC, the common symptoms associated with presentation related to sexual violence were mood- (46·6%) and anxiety-related (45·1%) symptoms. In Iraq, as well as anxiety and physical complaints, behaviour-related complaints were common in presentations associated with psychological violence, witnessing abuse or injury, domestic violence and non-violence-related incidents.

**Table 6:** Presenting complaint category for children and adolescents

	DRC	Iraq	OPT	Total	*P*-value*
Anxiety-related:Including PTSD-related symptoms such as flashbacks, avoidance, unwanted images/thoughts, fear/anxiety, intense psychological distress, hyper-vigilance, constant worrying)					
*n* (%)	348 (33·1)	639 (38·2)	96 (32·9)	1083 (35·9)	0·015
Mood-related:Including depression-related symptoms such as sadness, loss of interest/inability to experience pleasure from usually enjoyable activities (anhedonia), hopelessness, guilt,self-blame, feeling worthless, suicidal thoughts, suicidal intention/attempts)					
*n* (%)	341 (32·5)	170 (10·2)	33 (11·3)	544 (18·0)	<0·001
Behaviour-related:Including aggression, frequent conflicts, delinquent behaviour, impulsiveness, compulsive or repetitive behaviour)					
*n* (%)	46 (4·4)	204 (12·2)	25 (8·6)	275 (9·1)	<0·001
Physical:Including enuresis, general body pain, unclear single physical complaint/aches, unclear multiple physical complaints/aches, palpitations, weakness)					
*n* (%)	157 (14·9)	298 (17·8)	116 (39·7)	571 (18·9)	<0·001
Other					
*n* (%)	159 (15·1)	363 (21·7)	22 (7·5)	544 (18·0)	<0·001
Total					
*n* (%)	1051 (100)	1674 (100)	292 (100)	3017 (100)	

* Fischer’s Exact test.

**Table 7:** Commonly reported precipitating events and the associated most commonly reported presenting complaints by children and adolescents, by country

	*n* (%)	95% confidence interval
DRC:		
*Sexual violence*	384 (100)	–
Mood-related problems	179 (46·6)	41·5–51·7
Anxiety-related complaints	173 (45·1)	40·0–50·2
Physical complaints	15 (3·9)	2·2–6·4
*Non-violence*	316 (100)	–
Physical complaints	94 (29·8)	24·8–35·1
Mood-related problems	84 (26·6)	21·8–31·8
Anxiety-related complaints	41 (13·0)	9·5–17·2
Iraq:		
*Psychological violence*	298 (100)	–
Anxiety-related complaints	112 (37·6)	32·1–43·4
Physical complaints	49 (16·4)	12·4–21·1
Loss/mourning	36 (12·1)	8·6–16·3
Mood-related problems	35 (11·7)	8·3–16·0
*Witnessing abuse, injury or death*	242 (100)	–
Anxiety-related complaints	106 (43·8)	37·5–50·3
Physical complaints	39 (16·1)	11·7–21·4
Behaviour-related	31 (12·8)	8·9–17·7
Loss/mourning	28 (11·6)	7·8, 16·3
*Domestic discord or violence*	426 (100)	–
Anxiety-related complaints	162 (38·0)	33·4–42·8
Physical complaints	79 (18·5)	15·0–22·6
Family-related problem(s)	67 (15·7)	12·4–19·5
Behaviour-related	48 (11·3)	8·4–14·7
*Non-violence related*	286 (100)	–
Anxiety-related complaints	110 (38·5)	32·8–44·4
Physical complaints	54 (18·9)	14·5–23·9
Behaviour-related	38 (13·3)	9·6–17·8
Age-related problems	30 (10·5)	7·2–14·6
oPt:		
*Incarceration/detention*	94 (100)	–
Sleep problems	18 (19·2)	11·8–28·6
Enuresis	18 (19·2)	11·8–28·6
Anxiety/stress	8 (8·5)	3·7–16·1
Witnessed violence (physical, killing)	49 (100)	–
Anxiety/stress	10 (20·4)	10·2–34·3
Excessive fear/phobia/feeling threatened	9 (18·4)	8·8–32·0
Domestic violence	37 (100)	–
Enuresis	13 (37·1)	20·2–52·5
Anxiety/stress	6 (17·1)	6·2–32·0
Aggression	6 (17·1)	6·2–32·0
*Received threats*	32 (100)	–
Enuresis	10 (31·3)	16·1–50·0
Excessive fear/phobia/feeling threatened	4 (12·5)	3·5–29·0
Irritability/anger	4 (12·5)	3·5–29·0
Hypo-activity/hyper-activity (lack, loss or excess of energy)	3 (9·4)	2·0–25·0
Anxiety/stress	3 (9·4)	2·0–25·0

Not all percentages will total 100% because not all precipitating events are included.

In oPt, commonly reported symptoms associated with incarceration or detention were sleeping problems (19·2%) and secondary nocturnal enuresis (19·2%). Other common precipitating events in oPt were witnessing violence (associated with anxiety-related symptoms) and domestic violence and threats, both of which were associated most commonly with secondary nocturnal enuresis and anxiety-related symptoms.

### Service provision and outcomes

**Figure 2:**
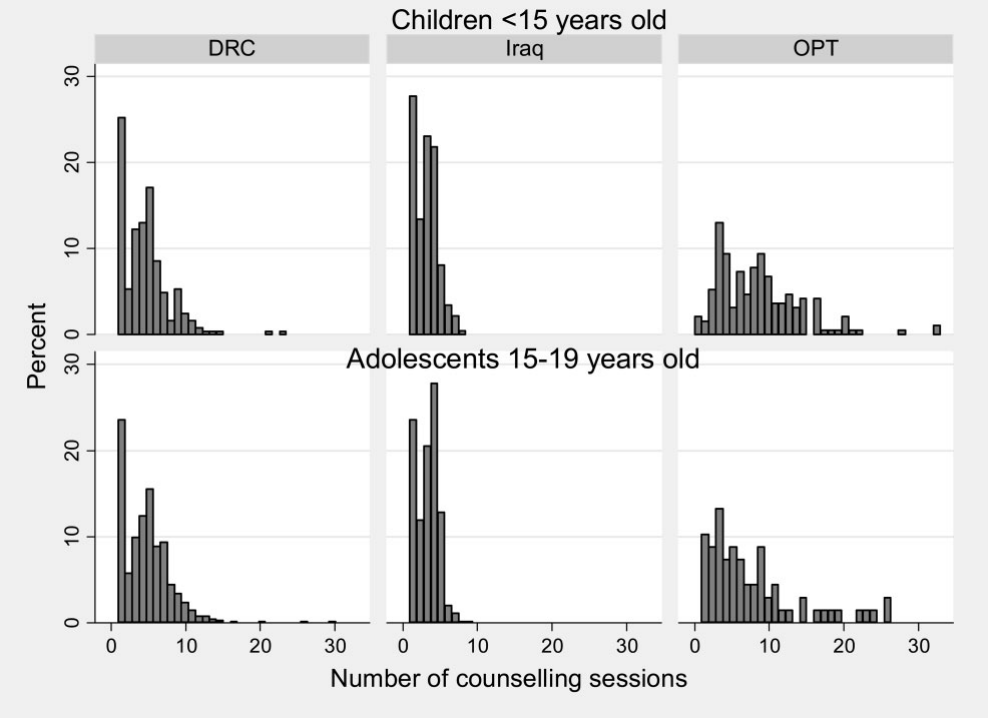
Number of counselling sessions by country for children and adolescents. DRC, the Democratic Republic of Congo; oPt, the occupied Palestinian territory

The number and distribution of counselling sessions received by each child or adolescent varied between countries (Figure 2). In DRC and Iraq, most children and adolescents received between two and five counselling sessions, and very few received more than 10 (4% in DRC and none in Iraq). In oPt, however, variation in the number of sessions was much greater, with 44% of children and 38% of adolescents receiving more than 10 counselling sessions.

A high proportion of children (47·2%) and adolescents (44·6%) defaulted before care was completed (Table 8). These proportions varied significantly by country for both age groups (40·6%, 54·5% and 29·6% of children and 37·9%, 49·7% and 50·6% of adolescents defaulting in DRC, Iraq and oPt, respectively, *P*<0·001 for both age groups).

**Table 8:** Proportion of children and adolescents discharged from care by country

	DRC	Iraq	oPt	Total	*P*-value*
Children, <15 y:					
Defaulted, *n* (%)^†^	112 (40·6)	419 (54·5)	63 (29·6)	594 (47·2)	<0·001
Discharged, *n* (%)	164 (59·4)	350 (45·5)	150 (70·4)	664 (52·8)	
Total, *n* (%)	276 (100)	769 (100)	213 (100)	1258 (100)	
Adolescents, 15–19 y:					
Defaulted, *n* (%)^†^	294 (37·9)	451 (49·7)	43 (50·6)	788 (44·6)	<0·001
Discharged, *n* (%)	481 (62·1)	456 (50·3)	42 (49·4)	979 (55·4)	
Total, *n* (%)	775 (100)	907 (100)	85 (100)	1767 (100)	
Children and adolescents combined, <19 y					
Defaulted, *n* (%)^†^	406 (38·63)	870 (51·91)	106 (35·57)	1382 (45·69)	<0·001
Discharged, *n* (%)	645 (61·37)	806 (48·09)	192 (64·43)	1643 (54·31)	
Total, *n* (%)	1051 (100)	1676 (100)	298 (100	3025 (100)	

* Fischer’s Exact test;

^†^ patient left programme before care completed.

**Figure 3:**
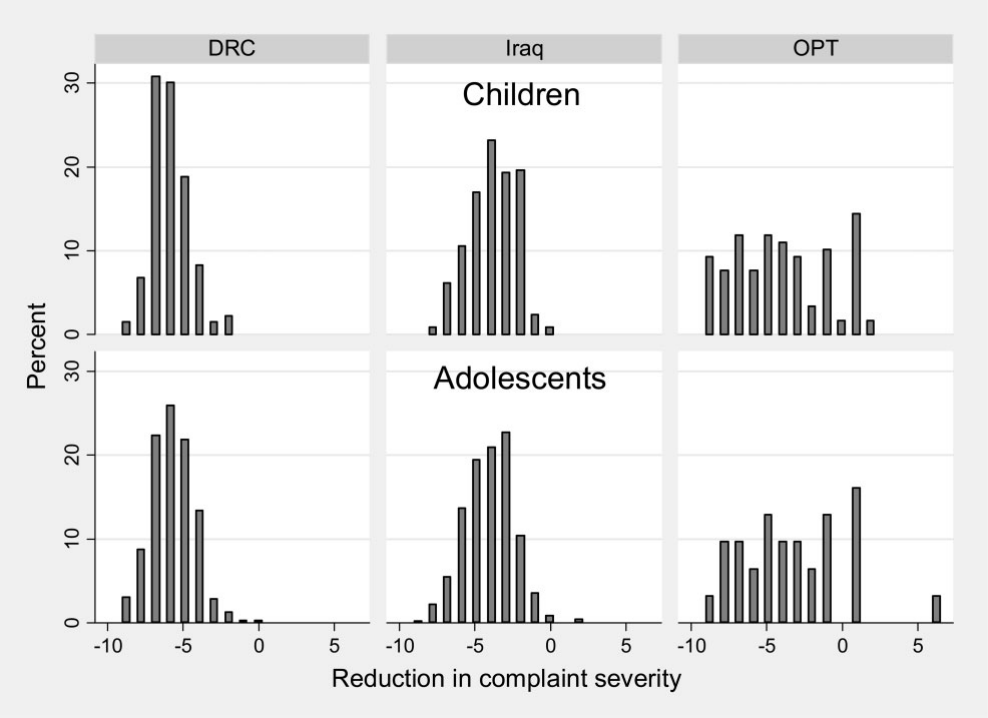
Change in presenting complaint-rating for those discharged from care, by age group and country. The severity of the presenting complaint is self-rated by the patient on a scale of 1–10 in all three countries; change in complaint severity is calculated by subtracting the rating at presentation from the rating at discharge. A negative difference indicates that the complaint decreased in severity between presentation and discharge. DRC, the Democratic Republic of Congo; oPt, the occupied Palestinian territory

Of children and adolescents discharged from care, 99·8%, 98·9% and 81·9% in DRC, Iraq and oPt, respectively, reported improvement of their presenting complaint following treatment (Table 9). As Figure 3 demonstrates, the magnitude of this change (i.e. reduction in severity of presenting complaint) varied between countries, with greater variation in change in oPt than in the other two countries for both children and adolescents.

**Table 9:** Status of presenting complaint (indicated by self-reported change in complaint rating) at completion of care of children and adolescents by country and exit type

	DRC	Iraq	OPT	Total
Total children and adolescents discharged:	645	806	192	1643
Of discharged, those with a rating at final visit	522	794	149	1465
% discharged with rating at final visit	80·9	98·5	77·6	89·2
Children discharged from care:				
Better, *n* (%)	133 (100)	338 (99·1)	97 (82·2)	568 (96·0)
The same, *n* (%)	0	3 (0·9)	2 (1·7)	5 (0·8)
Worse, *n* (%)	0	0	19 (16·1)	19 (3·2)
Total, *n* (%)	133 (100)	341 (100)	118 (100)	592 (100)
Adolescents discharged from care:				
Better, *n* (%)	388 (99·7)	447 (98·7)	25 (80·7)	860 (98·5)
The same, *n* (%)	1 (0·3)	4 (0·9)	0	5 (0·6)
Worse, *n* (%)	0	2 (0·4)	6 (19·4)	8 (0·9)
Total, *n* (%)	389 (100)	453 (100)	31 (100)	873 (100)
Children and adolescents leaving prior to discharge (defaulted):				
Total defaulting	406	870	106	1382
Of defaulters, those with a rating at final visit	190	319	24	533
% defaulting with follow-up rating with rating at final visit	46·8	36·7	22·6	38·6
Better, *n* (%)	150 (79·0)	287 (90·0)	16 (66·7)	453 (85·0)
The same, *n* (%)	38 (20·0)	31 (9·7)	6 (25)	75 (14·1)
Worse, *n* (%)	2 (1·1)	1 (0·3)	2 (8·3)	5 (0·9)

A complaint rating at the last recorded visit was available for 46·8%, 36·7% and 22·6% of those leaving care before discharge in DRC, Iraq and oPt, respectively (Table 9). Within these groups, 79·0%, 90·0% and 66·7% in DRC, Iraq and oPt self-reported improvement of the presenting complaint.

## Discussion

This paper presents descriptive information on the characteristics and presenting complaints of children and adolescents attending mental health services with varying approaches to service provision in three countries affected by armed conflict, and of the patterns and outcomes of care.

### Access to care

Children and adolescents represent a small proportion of patients presenting to these services, but the proportion varied significantly by country. It is important to note that Iraq and oPt, although more similar in terms of culture and conflict, had very different proportions of children and adolescents amongst those who presented. In fact, Iraq was more similar in this regard to DRC, suggesting that this difference was related more to programme characteristics than to context. Children also constitute a relatively small proportion of patients presenting to other MSF mental health programmes, although again there is considerable variation across programmes.^29^ Across all MSF-OCA mental health programmes in 2009, 12% of clients were under 18 years of age. The majority of these programmes were not in active armed conflict settings, suggesting that low presentation of children is not unique to armed conflict settings.

There is likely to be a proportion of children with mental health complaints who did not present to MSF services, and external barriers to access may not be the only factor associated with this. The existing literature suggests that children are likely to constitute a substantial proportion of those with psychological illness in conflict settings, including the countries in this analysis. A prevalence study in Mosul, Iraq documented a 37·4% point prevalence (40·2% and 33·2% for boys and girls, respectively) of mental disorders among children under 15 years of age.^20^ In a study of 959 Palestinian children aged 6–12 years in the Gaza Strip, the prevalence of behavioural and emotional problems was 54·5% in boys and 46·5% in girls.^30^ MSF experience in general is that children often constitute the majority of patients seen in other types of health services such as primary health care, even in conflict settings [personal communication]. This might suggest that mental health needs are not recognized or are seen as less important by parents and/or medical staff, or that health services are not considered an option for therapeutic interventions for childhood mental health problems. The limited literature on mental health services in conflict areas does also suggest that, despite the high prevalence of mental distress in children, there might be only a small proportion who require individual counselling.^31^ In a study using a multi-tiered mental health care package in several conflict-affected countries (Burundi, Sri Lanka, South Sudan, Indonesia and Nepal), the majority of children were provided with community and classroom-based interventions and only 6% of all children screened overall were referred for individual or family counselling. Our findings may partly reflect this tiered distribution of mental healthcare needs, where only a small proportion of children require individual counselling.

Specific provision of information on child mental health services, by providers of those services, to groups that come into contact with children appears to improve uptake. In oPt, children are one of the key target groups of the programme, and are identified through specific community outreach and networking activities. This may be one reason why the proportion of children in this programme is significantly higher than in the other programmes. In oPt, most patients were referred through active outreach and contact with MSF counsellors themselves, other organisations aware of the services or family members.

In DRC, the integrated nature of the programme and links with community health workers is reflected in the fact that almost half of all patients were referred by primary healthcare services, whereas in Iraq the focus on links to secondary and tertiary health services meant they were of more importance than primary care providers. Community outreach in DRC was also an important source of referral. This outreach by MSF includes specific information on sexual violence services, which may be a reason for the high proportion of survivors of sexual violence, including children, presenting to the programme.

The majority of presentations in oPt were male. Although girls were a minority of those presenting for care in this analysis, past community-based prevalence studies in oPt have found equal or higher levels of mental health impacts in girls.30,^32^ The difference between our service-based findings and these other community-based studies may be owing to lack of access to services for female children, or to different presentations for care according to type of exposure, amongst other reasons.

### Precipitating events

The type of underlying or precipitating event associated with patients presenting for care varied significantly across countries. In DRC, sexual violence was the main reason for presentation (40·7% of children), and, as the majority in this group were girls, this is reflected in the overall preponderance of females in the DRC programme. Violence against women in DRC, including sexual violence against girls, is extremely prevalent. One study found that 6% of survivors of rape presenting at a hospital in DRC were younger than 16 years.^33^ Researchers have noted that the psychological consequences of rape can be at least as devastating as the physical consequences.^33^^–^35 Very few children or adolescents presented for sexual violence in Iraq or oPt.

Armed conflict was the most common category of event associated with presentation in Iraq and oPt. In this category in Iraq, children and adolescents most commonly reported witnessing violence or being exposed to psychological violence, rather than being directly subjected to physical violence. In oPt, however, being directly subjected to detention or incarceration was the most common precipitating event reported by children and adolescents.

Domestic violence or neglect was a common precipitating event, reported by 21·4% and 16·4% of children in Iraq and oPt, respectively. Although little has been documented on children’s exposure to domestic violence in conflict settings, it is now increasingly recognised that levels of intimate partner violence increase during conflict;36,^37^ these two exposures are closely linked,^38^ and children in households with high levels of intimate partner violence face the indirect impact of this violence, as well being at greater risk of themselves suffering violence by a family member.39,^40^ Family violence also undermines a mother’s capacity to provide support, and undermines children’s trust in the perpetrator, decreasing their capacity to be resilient.^9^

Increased levels of intimate partner violence related to ongoing armed conflict has been reported in the literature from DRC,33,^41^^–^43 but the prevalence of domestic violence in DRC in our analysis was lower than in the other two settings. It might be that more domestic violence in DRC is sexual, and therefore was reported within that category in this analysis.

Another important finding, not unique to MSF experience, is that a considerable proportion of all presentations were not directly linked to violence.^44^

### Presenting complaints

Symptoms reported commonly in our analysis are consistent with the existing literature for those settings. In a cross-sectional study of children attending primary healthcare centres in Mosul, Iraq, the most prevalent mental health conditions included PTSD (10·5%), enuresis (6%), separation anxiety disorder (4·3%) and specific phobia (3·3%).^20^ Studies have demonstrated that Palestinian children face an increased risk of developing anxiety, depression and symptoms of PTSD, amongst other emotional and behavioural problems.45,^46^

The distribution of categories of symptoms varied between the three countries. Mood-related disorders were more frequent in DRC than in the other two, which might be attributable to the fact that mood-related disorders were commonly reported by those presenting after sexual violence, which was in turn the most commonly reported exposure in DRC. In a hospital-based study in DRC of patients presenting following rape, many of whom were children, survivors reported feelings of sadness, anger, fear, anxiety, shame and misery.^33^ Physical complaints, in particular enuresis, were most common in oPt.

Despite the unique contexts of armed conflict in which these exposures occurred, the symptoms commonly reported in this analysis are also those reported by children in other settings, including non-conflict settings and in developed countries.^47^ Additionally, these symptom profiles are similar to those reported by children and adolescents exposed to violence, domestic violence or sexual trauma in non-conflict settings.^48^

### Mental health outcomes

Almost half of all patients defaulted before discharge by a mental healthcare provider. High rates of default have been reported by mental health programmes in non-conflict settings; for example, a study of survivors of Hurricane Katrina found that, 8 months later, 60% of those commencing mental health programmes had dropped out of care.^49^ This suggests that default may not simply be explained by difficulties of access or population mobility related to conflict.

Although default rates were high, outcomes were generally good in terms of self-reported improvement of presenting complaints in those completing care, even in settings with non-specialised mental health staff. Follow-up ratings were available for only a minority of those who defaulted, so inferences on the outcomes of incomplete care cannot be drawn with any reliability.

### Limitations

This study presents data from three different settings and programmes with differing approaches to service delivery and provision of care, and therefore any comparison of outcomes, without taking account of these differences, might give rise to misleading conclusions.This paper reports findings from service data on patients presenting for care to these programmes. Many factors can influence access to and uptake of care and it is therefore not possible to assess the degree to which these findings quantitatively reflect the situation in the source communities from which these patients were drawn. In DRC and Iraq, the programmes were not specifically designed for children who constitute a relatively small proportion of patients in those programmes. Although outcomes were generally good in those who completed care, interpretation of findings must be qualified by the high rates of default in all settings. Additionally, as there was no reference or control group, improvement in presenting complaints cannot necessarily be attributed to the provision of care.

## Conclusion

Children and adolescents accounted for a small proportion of those presenting for mental healthcare in programmes not specifically targeting these age groups. Uptake by children and adolescents could be improved through the provision of child and adolescent-specific mental health service information, the use of community-based outreach activities, and linkage to other sectors of the health system to target such exposures.

In recognising the indirect impact of conflict on children, medical care has shifted from a narrow focus on treating only the war-wounded to providing comprehensive primary healthcare. A similarly comprehensive approach is required for the treatment of mental health issues, encompassing not only armed conflict-related presentations but other violence such as domestic violence and non-violence-related events. All three programmes included in this analysis utilised this approach.

Symptom profiles varied by context and precipitating event, and were similar to those reported by children and adolescents exposed to violence or sexual trauma in non-conflict settings. Brief trauma-focused therapy, the current preferred MSF mental health therapeutic intervention, appeared to be effective in reducing symptoms precipitated by various forms of trauma. This provides a feasible tool for addressing the mental health needs of children exposed to armed conflict.

The research agenda for mental health issues for children and adolescents is long. Priorities arising from this analysis include understanding why children and adolescents constitute a small proportion of patients in some programmes, the impact of exposure such as domestic violence, why many default and how to address these issues. However, this research must occur within the context of efforts to provide mental health services for children, which, as this paper demonstrates, is possible even under the constraints imposed by armed conflict.
